# Case reports: are they still useful?

**DOI:** 10.31744/einstein_journal/2026ED2691

**Published:** 2026-09-02

**Affiliations:** 1 Hospital Israelita Albert Einstein São Paulo SP Brazil Hospital Israelita Albert Einstein, São Paulo, SP, Brazil.

This journal publishes case reports^(1)^ when they: (a) report a previously undescribed disease or novel manifestations of a known disease; (b) describe outbreaks or atypical disease patterns; (c) document adverse treatment effects not yet widely recognized; (d) report the use of therapeutic agents not conventionally indicated for the condition under consideration; or (e) address therapeutically challenging situations for which no consensus-based management guidelines exist.

Some journals rarely accept case reports, while others publish them exclusively.^(2-4)^

A conventional threshold exists in the literature: reports describing six or fewer cases are classified as case reports, whereas series comprising more than six cases are typically considered original articles.^(5)^

Case reports must adhere to established ethical standards. Manuscripts must be reviewed and approved by the Institutional Review Board (IRB) of the submitting institution prior to submission. Written informed consent from the patient(s) is mandatory. When a submitting institution lacks an IRB, approval must be obtained from an affiliated institution with the appropriate oversight capacity. Patient confidentiality must be rigorously protected: identifying information should be removed, and photographs should, at minimum, obscure the eyes. We recognize that, in the setting of exceedingly rare conditions, complete anonymization may be challenging; nonetheless, authors are expected to take all reasonable measures to minimize the risk of patient identification.^(6)^

Case reports should be concise. Lengthy discussions or systematic literature reviews appended to reports of one or two cases rarely enhance the scientific contribution of the manuscript.

Case reports are scientific manuscripts and must provide sufficient information to enable independent assessment of the findings. All relevant data, including clinical, laboratory, and diagnostic results, must be reported and interpreted. Manuscripts lacking detailed laboratory data will not be considered for publication.

Educational case reports are among the most effective tools in medical education. Medical students demonstrate greater engagement when actively participating in clinical rounds and case-based discussions. Academic discourse structured around individual cases provides an integrated platform for examining the clinical, pathophysiological, and basic science dimensions of disease. Contemporary medical schools increasingly use case reports as pedagogical instruments to foster comprehensive clinical reasoning. In the context of continuing medical education, case reports serve as valuable tools for in-depth, disease-focused learning among practicing physicians.^(7,8)^

This journal is pleased to announce the launch of a new section, "Einstein Case Reports of *Hospital Israelita Albert Einstein*," in which complex clinical cases encountered at our institution will be presented and analyzed by our physicians, medical students, and fellows. These discussions will be modeled on the format of the Case Records of the Massachusetts General Hospital as published in the New England Journal of Medicine. Our institution includes a Medical School, and the education of medical students constitutes a core component of our institutional mission. In addition, we maintain an active continuing medical education program for our clinical staff. We are confident that this new section will serve as a valuable resource for all health professionals.

## Data Availability

Data are available to reviewers upon request.
